# Investigating Social Determinants of Health in an Urban Direct Primary Care Clinic

**DOI:** 10.7759/cureus.10791

**Published:** 2020-10-04

**Authors:** Leila C Tou, Nirmala Prakash, Shereen J Jeyakumar, Srekar Ravi

**Affiliations:** 1 Department of Internal Medicine, Charles E. Schmidt College of Medicine at Florida Atlantic University, Boca Raton, USA; 2 Department of Integrated Medical Science, Charles E. Schmidt College of Medicine at Florida Atlantic University, Boca Raton, USA

**Keywords:** direct primary care, social determinants of health, internal medicine, prapare

## Abstract

Direct primary care (DPC) is an emerging model of care distinguished by lower price points for quality comprehensive services. The affordability of DPC attracts a broad patient population that may encompass a wide range of socioeconomic needs. It is critical to identify social determinants of health (SDH) in DPC practices to design strategies aimed to mitigate social risk factors, especially for vulnerable populations that can only afford DPC. As part of this SDH screening initiative, the purpose of the present descriptive study was to assess the SDH characteristics of patients from an urban DPC clinic. To identify these SDH factors, a cohort of 31 patients from the DPC clinic was asked to complete a questionnaire from the Protocol for Responding to and Assessing Patient Assets, Risks, and Experiences (PRAPARE). The survey outcomes revealed top socioeconomic needs in the domains of stress (77.4%), insurance (51.6%), social integration and support (38.7%), unmet medicine or healthcare needs (35.5%), and unemployment (32.2%). In adopting a community-based participatory research (CBPR) approach, the research team shared the survey outcomes with the DPC clinic to facilitate improvements in overall patient care and implementation of services aimed to address social risk factors as identified in the study.

## Introduction

Individuals of low socioeconomic status (SES) suffer disproportionally poorer health outcomes compared to their more affluent counterparts [1]. This SES health gradient is contingent on a host of psychosocial, physical, and healthcare domains [1-2]. These domains or social determinants of health (SDH) impact a patient’s ability to attain opportunities and resources to protect, improve, and maintain health [3]. Among SDH factors, insurance status, or the lack thereof is by far the most common barrier to accessing healthcare [4]. In the United States, approximately 30.1 million persons under the age of 65 are uninsured, and 4.8% of persons cannot obtain medical care due to cost [5]. Direct primary care (DPC) employs a “contract practice” concept to address this barrier by expanding access to patients who cannot afford traditional insurance-based healthcare. The use of “contract practice” can be traced back to pre-Civil War U.S. medicine where patients paid fixed fees for comprehensive health services. Third-party payer systems like insurers were developed to prevent patients from using services beyond what is covered by the fixed fee [6]. Adopting a modified “contract practice” framework, DPC clinics define set primary care services for which their patients pay a monthly, quarterly, or annual flat fee (i.e. retainer fee) [6-7]. As a result, the patients bypass the traditional fee-for-service (FFS) model and restrictions placed by third party insurers [7]. DPC delivers cost-effective healthcare to a diverse socioeconomic population through a direct financial partnership between the patient and primary care provider.

Through “contract practice,” DPC eliminates administrative tasks and costs associated with third-party billing thereby allocating more time for meaningful patient-physician interaction. Administrative burden is one of the leading causes of stress and burnout for U.S. physicians [8]. On average, a physician spends nearly 25% of their time on non-clinical work such as coding, billing, and dealing with middleman insurance companies [9]. Preoccupation with administrative busywork and pressure to maintain financial sustainability means that healthcare providers spend less quality time with their patients [8-9]. These concerns have fueled many physicians to transition to the DPC model of care [9-10]. As a result, the integration of DPC promises reprieve from stress related to third-party billing while offering the opportunity to provide meaningful patient care.

The benefits conferred from the DPC model of care are likewise extended to patients. With reduced overhead administrative costs, DPC clinics can offer more affordable care. The average monthly DPC fee for adults is estimated to be $77.38 with a range of $42-$125.32 [7]. In contrast, the average cost per appointment for a new uninsured patient is $160 with a range of $128-$188 in the traditional insurance-based system [11]. These low DPC rates are fixed regardless of pre-existing health conditions and typically do not require additional copays or deductibles for comprehensive services [12]. Affordability is also due to decreased costs for prescriptions and ancillary services. Prescription medications are often dispensed at a lower cost or included as part of membership fees without an increase. For example, one DPC patient paid just $3 for a three-month supply of prescription medication that would have cost $40 monthly [12]. Many DPC clinics also partner with imaging centers and labs to provide ancillary services to their patients at a reasonable cost. This is demonstrated by a DPC clinic that charged $5 for prostate cancer tests that would typically cost more than $175 for a Medicare patient [12]. Another advantage of DPC is an improved patient experience. With less administrative work and pressure to see a high volume of patients, physicians can revitalize a value-based care system rather than a volume-focused one. A 2005 survey of 231 DPC physicians reported that a typical DPC practice maintains a patient panel of 898 whereas the typical traditional insurance-based practice panel consists of approximately 2,303 patients [6]. These smaller panel sizes contribute several advantages including enhanced accessibility and flexibility. Unlike patients in insurance-based healthcare, DPC patients frequently experience expanded 24/7 access to their physicians beyond the conventional office visit. DPC patients may contact their physicians through emails, text messages, phone calls, and visits to other locations (i.e. house calls) on an as-needed basis [13]. As a part of increased physician access, DPC patients benefit from same-day or next-day appointments compared to the typical slow turnarounds in appointment scheduling common to traditional primary care. On average, DPC appointments consist of 30-60 minutes [13] in contrast to the seven-minute meetings in insurance-based healthcare [13-14]. These benefits are particularly advantageous to low SES patients whose care is thwarted by sociodemographic factors and excessive healthcare costs but have a high risk of serious comorbidities that may require more attention [4]. Altogether, these benefits engender quality care with a decreased burden of cost for DPC patients.

Under DPC, physicians can provide personalized, comprehensive, and coordinated care. Stronger connections between patient and physician translate to better patient health outcomes. A study in the British Medical Journal reports that DPC patients experienced 35% fewer hospitalization, 65% fewer emergency department visits, 66% fewer specialist visits, and 82% fewer surgeries compared to patients enrolled in traditional primary care. DPC patients also experienced a 56% and 49% decrease in non-elective and avoidable admissions, respectively, where there was a 91-97% decrease in patient readmission for conditions such as acute myocardial infarction, pneumonia, and congestive heart failure [15]. Through increased quality patient interaction, DPC physicians effectively manage chronic conditions, medications, and lifestyle factors contributing to better patient outcomes.

The DPC model of care addresses the modern needs of both patients and physicians as demonstrated by a high reported overall satisfaction. Compared to their counterparts in the traditional healthcare system, DPC physicians reported a higher level of career satisfaction [16]. These statistics can be attributed to the restored physician autonomy and decreased risk of burnout in DPC as discussed previously [8,16]. Patients in the DPC network also reported contentment with the model of care. One study placed the overall satisfaction for patients in one DPC clinic at the 95th percentile, likely owing to the cost-effectiveness, unique patient-physician dynamics, and positive health outcomes in DPC. This is a stark contrast to the drawbacks of traditional primary care where patient satisfaction is at the 90th percentile [17]. An unpublished study by Jeyakumar et al. explored patient perceptions of joining a DPC clinic (Poster: Jeyakumar SJ, Ravi SN, Prakash N. A Community-Based, Qualitative Assessment of a Direct Primary Care Practice: A Pilot Study. Florida Atlantic University Schmidt College of Medicine Medical Student Research and Scholarship Day. February 28, 2020). In the study population, patients turned to DPC due to poor past healthcare experiences (23.5%) and the inability to afford health insurance (9.6%). DPC caters to a specific demographic of disillusioned patients who feel that they “fell through the cracks” of traditional primary care. The barriers in traditional primary care faced by both physicians and patients alike compel them to join DPC.

DPC clinics care for a diverse socioeconomic patient population including the uninsured and underinsured. As a result of affordability and increased access to DPC, it becomes important to understand SDH domains that underlie poor clinical outcomes in vulnerable populations that rely on DPC for care. Screening of SDH characteristics can help identify the breadth and scope of these barriers to health in DPC patient panels. Several validated SDH assessment tools have been developed including the standardized questionnaires from the National Academy of Medicine (NAM) and Protocol for Responding to and Assessing Patients' Assets, Risks, and Experiences (PRAPARE) [18]. These SDH screening tools have been employed in previous studies including the Kusnoor et al. cross-sectional study [18], Gold et al. pilot study [19], and Weir et al. study [20]. Kusnoor et al. used a hybrid survey composing of questions from NAM, PRAPARE, and Survey of Household Economics and Decisionmaking (SHED) to assess the social and behavioral determinants of health (SBDs) in 100 patients from Connectus Health Vine Hill, an urban community health clinic [18]. Gold et al. also adopted a hybrid questionnaire incorporating questions from NAM and PRAPARE to investigate SDH domains in 1,130 patients in three Pacific Northwest community health centers (CHC) [19]. Weir et al. implemented the PRAPARE survey in three cohorts across seven health centers [20]. All three studies identified a ubiquity of social risks in the CHC setting. This information may dictate how the surveyed health centers design strategies aimed to address these SDH factors such as programs that connect patients with community and public health resources.

The PRAPARE screening tool was developed by the National Association of Community Health Centers (NACHC) and its partners as part of a public health initiative to identify and address social determinants of health (SDH) [21]. The “Gold Standard” Stages of Measure Development were used for PRAPARE validation [21] in which (1) developers employed interview, environmental scans, and evidence-based literature to define constructs; (2) multi-stakeholder groups (i.e. health centers, health center networks, national brain trust groups, literacy experts, Primary Care Associations) participated in its development; (3) pilot studies were performed on approximately 3,000 patients; (4) ease of use and clarity of questions were examined through cognitive testing; (5) pilot results guided refinement; (6) SDH characteristics were assessed in a broad range of patient populations using the survey; (7) several validity and reliability tests (i.e. Greatest Lower Bound, Cronbach’s Alpha, Known Groups Validity) demonstrated good to excellent validity; (8) psychometric properties of PRAPARE are continuously evaluated and reported in presentations and conferences. PRAPARE has been increasingly used nationwide to assess SDH factors where data on its validity continues to grow.

While previous studies have assessed SDH domains in the community clinic setting, there is currently no published study evaluating SDH factors in a DPC setting to date. The purpose of the present descriptive study was to evaluate social risks in an urban DPC clinic from the Southeastern United States using the validated PRAPARE assessment tool to design strategies that address healthcare disparities and advance patient outcomes within the DPC clinic. Given the lower price points typical of DPC practices, it was hypothesized that the study population would comprise mostly uninsured patients with a high percentage of unemployment and many unmet needs (i.e. food, utilities, housing).

## Materials and methods

The present descriptive study adopted a community-based participatory research (CBPR) framework in collaboration with a DPC clinic located in the Southeastern United States. Here, the research team evaluated the SDH characteristics of patients in a typical DPC practice using the Protocol for Responding to and Assessing Patients' Assets, Risks, and Experiences (PRAPARE) survey. As part of the transdisciplinary CBPR approach, the results were shared with the DPC clinic to provide a better glimpse of the underlying factors that affect patient wellness. This study was approved by Florida Atlantic University Social, Behavioral and Educational Research Institutional Review Board (IRBNET ID #: 1395609-2).

Context

The research team partnered with an internal medicine DPC organization that provides comprehensive primary and preventative healthcare services to a diverse socioeconomic community. The practice first opened its doors in 2016 as one of the first to employ the DPC model in the region. It serves an urban community of 112,118 residents where the median household income is $47,764. Uninsured persons under the age of 65 account for 25% of the population. The population demographic is 60.9% White, 32.9% Black/African American, 0.2% American Indian and Alaskan Native, 1.3% Asian, 3.1% two or more races, and 20.8% Hispanic or Latino. Veterans account for approximately 6,200 of the population (5.5%) [22]. The size of the DPC patient panel at the time of the study was 75. 

Recruitment

The patients from the DPC clinic were recruited with the following inclusion criteria: participants must be 18 years or older and demonstrate an affiliation with the practice where a staff member identified them as a member to whom they provide healthcare services. The research team worked with the practice in creating an ethical recruitment strategy where participants were assured that their willingness to participate would not impact their quality of care and could opt out at any time. The clinic first handpicked willing participants based on past participation in focus groups. These participants were contacted and recruited using a standardized email and phone script. A flyer was also placed in the office to provide more information regarding the study to other potential participants. Of the 75 patients in the DPC practice, 31 patients (41.3%) were recruited to participate in the study. 

SDH screening tool

The 31 DPC patients were screened for SDH characteristics via the PRAPARE survey. Participants were asked to complete the questionnaire either in person during their office visits or online through email using a protected software called REDCap. Survey data were collected and managed using secure REDCap electronic data capture tools [23-24]. The PRAPARE survey assessed patient characteristics and SDH domains including gender, ethnicity, race, English proficiency, farm worker status, veteran status, income, housing situation, housing stability, household size and dependents, education, employment, insurance, social integration and support, transportation, stress, and material security [21]. The research team also included an additional question assessing the length of clinic membership. In total, the survey contained 19 questions that assessed patient characteristics and SDH domains. These responses were recorded on REDCap where descriptive analysis (i.e. frequency, percentages, charting) was performed. 

## Results

Patient characteristics

The present study demonstrated a diverse group of patients in the internal medicine DPC clinic (Table 1). The cohort consisted of 31 DPC patients who were surveyed using the PRAPARE tool where the following demographics were noted. Almost half of the participants were patients of the clinic for at least one year. The majority of the participants were male (71.0%) while females only represented 22.6% of the cohort. Participants were Asian (3.2%), Black/African American (35.5%), American Indian/Alaskan Native (3.2%), White (48.4%), more than one race (3.2%), and Hispanic or Latino (19.4%). A few respondents (16.1%) preferred speaking a language other than English. Lastly, the survey revealed that 6.5% of participants were farm workers and 12.9% were veterans. There was at least one participant who left a blank response or picked "I choose not to answer" for four of the seven domains evaluating patient characteristics. 

**Table 1:** Patient Characteristics The Protocol for Responding to and Assessing Patient Assets, Risks, and Experiences (PRAPARE) survey assessed patient demographics across several domains: gender, ethnicity, race, language preference, farm worker status, veteran status, and the length of membership at the clinic.

Characteristics	Percent of Participants
1) Gender:	
Male	71.0%
Female	22.6%
Other	0.0%
Chose Not To Answer/No Response	6.5%
2) Ethnicity:	
Hispanic or Latino	19.4%
Not Hispanic or Latino	80.6%
Chose not to answer/No Response	0.0%
3) Race:	
Asian	3.2%
Pacific Islander	0.0%
White	48.4%
Native Hawaiian	0.0%
Black/African American	35.5%
American Indian/Alaskan Native	3.2%
Other	6.5%
More Than One Race	3.2%
Chose Not To Answer/No Response	0.0%
4) Language Preference:	
English	83.9%
Language Other Than English	16.1%
Chose Not To Answer/No Response	0.0%
5) Farm Worker Status:	
Yes	6.5%
No	90.3%
Chose Not To Answer/No Response	3.2%
6) Veteran Status:	
Yes	12.9%
No	80.6%
Chose Not To Answer/No Response	6.5%
7) Length of Membership at Clinic:	
Less Than 6 Months	12.9%
6-12 Months	41.9%
1-2 Years	29.0%
2-3 Years	9.7%
3-4 Years	3.2%
Chose Not To Answer/No Response	3.2%

SDH domains

In addition to patient demographics, SDH domains were assessed in the same cohort (Table 2). The survey outcomes revealed that 77.4% obtained more than a high school education and 16.1% had a high school diploma or a GED (General Education Development). More than a quarter of respondents (32.2%) were unemployed and 3.2% had a part-time or temporary job. The median reported total annual household income (n=16) for the cohort was $52,500. Seasonal/migrant farm work was the main source of family income for 6.5% of participants. The median household size and number of dependents was one. Using the annual household income, the percentage of the federal poverty level (%FPL) was generated revealing 12.5% and 87.5% of the 16 respondents were at or below 200% FPL or above 200% FPL, respectively. Many of the respondents (64.5%) had household dependents either under the age of 18 (12.9%) or over the age of 65 (9.7%). Several participants provided invalid responses for questions assessing household size and the number of dependents. When asked about household size, 9.7% of respondents failed to include themselves in the count and answered "zero." When asked "how many people in your household depend on your income?", 6.5% of respondents answered "all" failing to provide a numeric value. 

**Table 2:** SDH Domains The Protocol for Responding to and Assessing Patient Assets, Risks, and Experiences (PRAPARE) questionnaire screened for social determinants of health domains such as housing status, housing stability, household size and dependents, education, employment, income, insurance, material security, transportation needs, social integration and support, and stress level. SDH: social determinants of health, CHIP: Children's Health Insurance Program, GED: General Educational Development, IQR: interquartile range, FPL: federal poverty level

Domain	Percent of Participants
1) Housing Status:	
Has Housing	80.6%
No Housing	6.5%
Chose Not To Answer/No Response	12.9%
2) Housing Stability:	
Worried About Losing Housing	22.6%
Not Worried About Losing Housing	58.1%
Chose Not To Answer/No Response	19.4%
3) Household Size and Dependents:	
Median Household Size (Including Participant)	One Person
Median Number of Dependents	One Person
Participants With Dependents Under the Age of 18 Years	12.9%
Participants With Dependents Above the Age of 65 Years	9.7%
4) Education:	
Less Than High School Degree	3.2%
High School Diploma or GED	16.1%
More Than High School	77.4%
Chose Not To Answer/No Response	3.2%
5) Employment:	
Unemployed	16.1%
Part-time or Temporary Work	3.2%
Full-Time Work	54.8%
Otherwise Unemployed But Not Seeking Work	16.1%
Chose Not To Answer/ No Response	9.7%
6) Income (n=16):	
Median Household Income (IQR)	$52,500 ($40,500-$76,625)
At or Below 200% FPL	12.5%
Above 200% FPL	87.5%
7) Insurance:	
None/Uninsured	51.6%
Medicaid	3.2%
CHIP Medicaid	0.0%
Medicare	6.5%
Other Public Insurance (not CHIP)	0.0%
Other Public Insurance (CHIP)	3.2%
Private Insurance	29.0%
Chose Not To Answer/No Response	6.5%
8) Material Security:	
Food	6.5%
Utilities	9.7%
Medicine or Healthcare	35.5%
Phone	3.2%
Clothing	6.5%
Child Care	0.0%
Other	6.5%
No Unmet Needs	48.4%
Chose Not To Answer/No Response	9.7%
9) Transportation:	
Lack of Transportation Affected Medical Appointments/Medicine	9.7%
Lack of Transportation Affected Non-Medical Meetings/Appointments	6.5%
Transportation Has No Affected Meetings/Appointments	71.0%
Chose Not To Answer/No Response	12.9%
10) Social Integration And Support:	
Less Than Once a Week	9.7%
1-2 Times Per Week	16.1%
3-5 Times Per Week	12.9%
More Than 5 Times Per Week	51.6%
Chose Not To Answer/No Response	9.7%
11) Stress:	
Not At All	16.1%
A Little Bit	12.9%
Somewhat	19.4%
Quite A Bit	29.0%
Very Much	16.1%
Chose Not To Answer/No Response	6.5%

The questionnaire also examined access to resources (Table 2). Of the 31 respondents, 6.5% did not have housing, and 22.6% were worried about losing their housing. In addition to housing needs, 16.1% had unmet transportation needs and 28.6% had at least one other unmet need (i.e. food, clothing, utilities). Of these needs, access to medical care was the most common response (35.5%) where there was a lack of insurance coverage. Most were uninsured (51.6%) while others were covered by Medicaid (3.2%), Medicare (6.5%), and other public insurance such as Children’s Health Insurance Program (CHIP) (3.2%). More than a quarter of participants (29.0%) were privately insured. Screening for social integration and support indicated that 38.7% of respondents interacted with their friends and family at most five times per week. A majority of respondents (77.4%) reported experiencing some level of stress. There was at least one participant who left a blank response or picked "I choose not to answer" for every question assessing SDH domains.

## Discussion

As the advantages associated with DPC become more apparent, the interest in adopting its model continues to grow. The American Academy of Family Practice conducted a large-scale survey with more than 20,000 U.S. physicians of whom approximately 7% reported that they adopted the DPC model and 13% are considering a transition in that direction [25]. As demonstrated by its increasing utility nationwide, the DPC framework is promising but its quality of patient care can be further advanced by recognizing the contributing role of SDH domains in poor health outcomes. 

SDH screening compliments standard diagnostic tools to capture a comprehensive picture of the patient from a social, psychological, and medical viewpoint. Identifying social and behavioral risks can inform strategies that aim to address prevalent socioeconomic needs in the community. These SDH-driven interventions can occur at the non-clinical, clinical, and community levels [21]. As part of this SDH screening initiative, the present descriptive study revealed top need areas among the DPC patients who were surveyed, including stress, insurance, social integration and support, unmet medicine or healthcare needs, and unemployment. Survey results from the present study were shared with the DPC clinic to tailor possible interventions to ameliorate the identified social risks.

The top SDH characteristic in the DPC cohort was stress. A majority of respondents reported experiencing some level of stress (77.4%) (Table 2). More than a quarter (29.0%) of patients reported feeling quite a bit of stress and 16.1% reported experiencing a lot of stress (Table 2). Chronic stress can contribute to a wide range of negative health consequences including the development of chronic health conditions such as high blood pressure, heart disease, and diabetes [26]. The National Association of Community Health Centers (NACHC) identified other potential social risks associated with patient stress including acts of self-harm, substance abuse, addiction, and sleep deprivation [21]. The stress component of SDH screening can inform clinicians of patient stress levels to facilitate improvements in stress management interventions. In the clinical setting, stress management interventions can focus on identifying stressors and providing healthy strategies to alleviate stress. Clinicians can also introduce patients to counseling services and social groups to reduce adverse health effects associated with stress [21]. 

Responses from the DPC participants revealed an unmet need in medicine or healthcare (35.5%) where there was a lack of insurance coverage (51.6%) (Table 2). The DPC cohort consisted of more than double the number of uninsured patients under the age of 65 in the community it serves (25%). The lack of insurance coverage in the DPC clinic is telling especially when viewed against the backdrop of uninsured patients nationwide. The Benchmark Survey is a nationally representative survey with roughly 3,500 respondents that examine insurance status. According to the Benchmark Survey, the number of uninsured participants in the DPC clinic surpassed the average uninsured patient share (5.7%) in internal medicine practices. The other 94.3% consists of patients who rely on Medicare (38.0%) Medicaid (11.9%), and Commercial Health Insurance (40.4%) [27]. In contrast, insured respondents of the present study relied on Medicaid (3.2%), Medicare (6.5%), other public insurances such as CHIP (3.2%), and private insurance (29.0%). This data suggested that the DPC clinic may be addressing a need for healthcare providers in its community who can offer care to underinsured or uninsured patients who experience more difficulty accessing quality care. Inadequate insurance coverage can impact a patient’s continuity of care and overall well-being [21]. It can be associated with other potential risks including the inability to afford medication and the increased use of hospital emergency rooms. Identifying a need for healthcare access may aid in structuring more affordable patient care. Clinicians can assess a patient’s knowledge of insurance enrollment processes and connect them with community healthcare workers, social services, and eligibility assistance workers as needed. To alleviate the financial barriers associated with the lack of insurance coverage, clinicians may also refer patients to generic medications [21]. 

The survey outcomes also demonstrated a lack of social integration and support (38.7%) in the DPC cohort. DPC patients responded that they interacted with friends and family less than once a week (9.7%), one to two times per week (16.1%), and three to five times per week (12.9%) (Table 2). As a factor that contributes to patient health outcomes, social support is another SDH domain that clinicians may evaluate to have a more comprehensive understanding of their patients. For example, social isolation can result in decreased immune function as well as increased neuroendocrine and cardiovascular activity [21]. In contrast, increased social integration has been associated with better health outcomes such as post-myocardial infarction prognosis [28]. Potential needs associated with inadequate social integration include a decreased likelihood of engaging with healthcare providers and a lack of knowledge about readily available resources that can address social isolation [21]. Screening can help clinicians integrate the needs of a patient including the need for social support into the care plan, which may incorporate connecting the patient to community resources or social services. 

The last area of need revealed from the survey responses was employment. The survey revealed that 32.2% of respondents were unemployed (Table 2). Of the 32.2% of unemployed respondents, 16.1% were not seeking work (Table 2). Employed respondents had a part-time job (3.1%), temporary work (54.8%), or a full-time job (16.1%) (Table 2). The median household income (n=16) was calculated to be $52,500 where the majority (87.5%) were above 200% FPL (Table 2). Limitations associated with surveys including nonresponse bias and voluntary response bias may have influenced the assessment of household income and percentage of the federal poverty level. Nevertheless, unemployment can impact many aspects of a patient’s life beyond the loss of a stable income. Without a good-paying job, patients may lack access to employee benefits, education, childcare services, and more nutritious food [21]. Facing multiple barriers to health, unemployed patients are at a higher risk of developing depression, suffering from stress-related conditions, and experiencing poorer health outcomes. Other potential needs associated with unemployment include the lack of healthcare coverage, inaccessibility to healthcare, and difficulties finding a job [21]. SDH screening can provide more insight into patients’ employment statuses to aid clinicians in responding to the needs of their patients accordingly. To reduce the impact of unemployment on patient health, clinicians may screen for mental health disorders, assess levels of stress, refer patients to employment centers for assistance, and provide community resources that aid in job searching.

The impact of social and behavioral factors on patient wellness necessitates clinicians to provide services to meet the needs of their patients. Given that SDH factors do not act in isolation, comprehensive interventions may be needed to effectively mitigate the adverse health impact associated with these social risks [21]. While not all organizations can provide comprehensive services to address socioeconomic needs, SDH interventions can be interdisciplinary by building community and inter-professional partnerships as discussed previously in the examples of services aimed to address the top needs identified in this study.

The first step in addressing socioeconomic needs is to identify them which was performed in this study using the PRAPARE survey. While the PRAPARE questionnaire remains a useful SDH screening tool, the survey difficulties experienced in the present study suggested that PRAPARE may require refinement before widespread implementation. First, PRAPARE has limitations that are common to surveys, including nonresponse and voluntary response bias as was discussed previously. There was at least one participant who left a blank response or picked “I choose not to answer” for the majority of the questions. Only 16 of 31 participants chose to provide information regarding their income (Table 2). Second, several invalid responses were received. For example, 6.5% of participants provided invalid responses when asked how many individuals in the household depend on their income, suggesting the question lacked clarity. In assessing the number of residents within the patients’ households, 9.7% of participants did not include themselves in the count although the question explicitly instructed them to do so. Invalid answers associated with household size and the lack of responses for questions assessing income led to difficulties in calculating the %FPL for each participant. The high rate of errors in responses and the lack thereof suggested that PRAPARE questions may require clearer language.

In addition to the aforementioned difficulties associated with the PRAPARE questionnaire, there were several limitations to the present study. First, several limitations common to descriptive studies include the lack of a control arm as well as the inability to test for statistical associations and hypotheses. Second, this study was founded upon self-reported data from a small DPC cohort that was recruited based on locational convenience. As a result of convenience sampling, the study population did not adequately represent a national demographic as there were disproportions in patient characteristics such as gender, race, and ethnicity (Table 1). In this regard, the assessment of SDH characteristics from this limited study did not apply to all urban DPC practices across the United States. Rather, the findings served to inform SDH-driven implementation tailored specifically to the DPC clinic investigated in this study where the sample population consisted of 41.3% of the total patient panel in the DPC clinic. Nonetheless, the present study is the first of its kind to examine social risk factors using a standardized SDH screening tool in an urban DPC setting to date. As such, this descriptive study may lay the foundation for more rigorous studies where large-scale sample populations with a nationally representative demographic are recruited to assess general patterns in SDH factors for DPC patients. Lastly, future studies may also focus on how to connect patients with appropriate resources and assess the efficacy of SDH-driven programs in addressing patient needs.

## Conclusions

DPC has the capacity to benefit both physicians and patients alike. For physicians, this model eliminates the insurance middleman and stabilizes finances. Rather than worrying about coding and billing, physicians will have time to prioritize the individual needs of their patients and build a strong rapport with them. Consequently, patients achieve more accessibility to quality routine, chronic, and preventive care services without being blindsided by additional financial barriers. As DPC is increasingly adopted by both clinicians and patients, clinicians may adopt routine SDH screening in their DPC clinics to further advance patient care. With new important insight into the SDH characteristics of their patient panel, DPC clinicians can appropriately tailor care to the needs of their patients. 
